# Correlation of weight and body composition with disease progression rate in patients with amyotrophic lateral sclerosis

**DOI:** 10.1038/s41598-022-16229-9

**Published:** 2022-08-02

**Authors:** Jin-Yue Li, Xiao-Han Sun, Zheng-Yi Cai, Dong-chao Shen, Xun-Zhe Yang, Ming-Sheng Liu, Li-Ying Cui

**Affiliations:** 1 Department of Neurology, Peking Union Medical College Hospital, Chinese Academy of Medical Science & Peking Union Medical College, Beijing, China; 2 Neuroscience Center, Chinese Academy of Medical Sciences, Beijing, 100730 China

**Keywords:** Neuroscience, Biomarkers, Diseases, Neurology

## Abstract

This study aims to observe the nutritional status of Chinese patients with amyotrophic lateral sclerosis (ALS), further investigating its effect on disease progression. One hundred consecutive newly diagnosed ALS patients and fifty controls were included. Weight and body composition were measured by bioelectrical impedance analysis at baseline and follow-ups. The revised ALS functional rating scale (ALSFRS-R) was used to calculate the rate of disease progression. Patients with ALS had a significantly lower BMI than controls, while no significant difference was found in body composition. Weight loss occurred in 66 (66%) and 52 (67.5%) patients at diagnosis and follow-up, respectively. Patients with significant weight loss (≥ 5%) at diagnosis had significantly lower BMI, fat mass (FM), and FM in limbs and trunk than those without. Fat-free mass (FFM), FM, and FM in limbs were significantly decreased along with weight loss at follow-up (p < 0.01). Patients with lower visceral fat index, lower proportion of FM, and higher proportion of muscle mass at baseline progressed rapidly during follow-ups (p < 0.05). Multivariate linear regression showed that FFM and weight at follow-up were independently correlated with disease progression rate at follow-up (p < 0.05). Weight loss is a common feature in ALS patients, along with muscle and fat wasting during the disease course. Body composition may serve as a prognostic factor and provide guidance for nutritional management in ALS patients.

## Introduction

Amyotrophic lateral sclerosis (ALS) is a neurodegenerative disease characterized by progressive dysarthria, limb weakness, and atrophy. Patients usually die of respiratory failure and nutrition dysfunction several years after onset^1^. Weight loss and malnutrition are common in patients with ALS, which suggest a poor prognosis and increased mortality^2,3^. Various factors are considered responsible for this condition. It is well established that dysphagia in ALS is linked to reduced dietary intake and weight loss^4^, while recent studies also suggested that loss of appetite^5^ and taste changes^6^ may also be involved in dietary changes. Additionally, evidence from clinical research revealed the presence of hypermetabolism in ALS patients^7^. Although controversial, a large number of clinical studies found an association between hypermetabolism and a faster rate of progression and shorter survival^8,9^.

Given the difficulty in the development of an effective treatment, metabolism exploration and management of nutrition in ALS patients has generated great interest in recent years. Until now, the nutritional state of patients with ALS has mostly focused on weight, body mass index (BMI), waist-hip ratio (WHR) and some serum nutritional biomarkers^10^. A few studies of body composition in ALS have showed different pattern of changes in body composition among ALS patients, focusing on the fat-free mass (FFM) and fat mass (FM)^3,5,11^. Also, some researchers suggested that muscle wasting was associated with poor prognosis, while gaining fat might be associated with better survival in ALS patients^3,12,13^. Accordingly, targeting nutritional status and supplementation of nutrients might be a promising therapeutic approach.

In this study, we aimed to evaluate the nutritional status of Chinese ALS patients by exploring weight and body composition during the course of disease and to explore the correlation between nutritional parameters and clinical parameters. Furthermore, the effect of nutritional status on rate of disease progression was also studied to identify the potential prognostic role of nutritional status in ALS.

## Results

A total of 100 newly diagnosed ALS patients (50 men and 50 women) and 50 normal controls were included in this study. The mean age at onset of patients was 51.90 ± 10.53 years, with a median (interquartile range) disease duration of 13 (8, 19) months at diagnosis. Seven patients had a positive family history and the bulbar form of onset was present in 35 (35%) ALS patients. The clinical characteristics of patients with ALS and the comparison of body composition between patients and controls are shown in Table 1. There were no significant differences in gender and age between patients and controls. The BMI was significantly lower in patients with ALS than in controls (p < 0.05), while no significant difference was found in weight, FFM, FM, and other nutritional parameters between the two groups.

**Table 1:** Clinical features of patients and the comparison of nutritional parameters between patients and controls.

	Patients (n = 100)	Controls (n = 50)	Effect size	95% CI	P value
**Demographics**					
Age at onset (years)*	51.90 ± 10.53				
Age at test (years)*	53.44 ± 10.22	54.64 ± 14.73	− 1.200^a^	− 5.824, 3.424	0.607
Sex (male)	50 (50%)	20 (40%)	1.78^b^	0.89, 3.57	0.247
**Clinical features**					
Duration (months)^§^	13 (8, 19)				
Bulbar onset	35 (35%)				
Predominance of LMN dysfunction	63 (63%)				
MRC sum score*	133.97 ± 25.81				
ALSFRS-R^§^	38 (32, 42)				
ALSFRS-bulbar scores^§^	10 (8, 12)				
ALSFRS-respiratory scores^§^	12 (10, 12)				
**Anthropometric measures**					
Weight (kg)*	65.25 ± 12.03	68.01 ± 12.14	− 2.77^a^	− 6.89, 1.36	0.19
BMI (kg/m^2^)*	23.52 ± 3.11	24.75 ± 3.34	− 1.23^a^	− 2.32, − 0.13	**0.028**
Fat-free mass (kg)*	47.83 ± 9.86	49.00 ± 9.39	− 1.17^a^	− 4.49, 2.15	0.487
Muscle mass (kg)*	44.70 ± 9.34	45.80 ± 8.88	− 1.10^a^	− 4.25, 2.05	0.49
Fat mass (kg)*	17.42 ± 5.04	19.01 ± 5.45	− 1.59^a^	− 3.37, 0.18	0.078
Bone mass (kg)*	3.14 ± 0.53	3.20 ± 0.50	− 0.06^a^	− 0.24, 0.12	0.488
MM%	68.46 ± 6.07	67.37 ± 5.46	1.09^a^	− 0.92, 3.10	0.286
FM%	26.72 ± 6.41	27.90 ± 5.78	− 1.18^a^	− 3.31, 0.94	0.274
Waist hip rate*	0.90 ± 0.06	0.89 ± 0.05	0.01^a^	− 0.01, 0.03	0.513
Visceral fat index*	9.79 ± 2.28	9.89 ± 2.12	− 0.10^a^	− 0.86, 0.66	0.798

*BMI* Body Mass Index, *ALSFRS-R* ALS Functional Rating Scale, *LMN* lower motor neuron, *MM%* proportion of muscle mass, *FM%* proportion of fat mass.

*Data presented as mean ± standard deviation.

^§^Data presented as median (IQR).

^a^Mean difference.

^b^Odd ratio.

P-value < 0.05 is shown in bold.

### Weight and body composition at diagnosis

The mean weight of ALS patients at diagnosis was 65.25 ± 12.03 kg, which was significantly lower than the premorbid weight (68.70 ± 13.39 kg, p < 0.001). Comparing with weight before disease onset, the median weight variation rate at diagnosis was − 4.11 (− 9.32, 0.56) %, and the median BMI variation was − 0.97 (− 2.42, 0.13) kg/m^2^. Of the 100 ALS patients included in this study, 4% were underweight (BMI < 18.5 kg/m^2^), 39% were overweight (BMI: 24–27.9 kg/m^2^) and 7% were obese (BMI ≥ 28 kg/m^2^). Compared with their premorbid weight, 66% had weight loss at diagnosis; among these patients, 65.2% (43/66) had a weight loss exceeding 5%, and 34.8% (23/66) had a weight loss exceeding 10%. According to their weight loss at diagnosis, the patients were divided into two groups: patients with significant weight loss (≥ 5%, n = 43); patients without significant weight loss (no weight loss or weight loss < 5%, n = 57). A comparison of clinical features and nutritional parameters between patients grouped by weight variation is shown in Table 2.

**Table 2:** Demographic, anthropometric and clinical features in different groups stratified by weight changes.

	Insignificant weight loss (n = 57)	Significant weight loss (n = 43)	Effect size	95% CI	P value
**Demographics**					
Age at onset (years)*	50.86 ± 10.15	53.28 ± 10.98	− 2.42^a^	− 6.63, 1.79	0.257
Sex			0.78^c^	0.35, 1.73	0.545
Male	27 (47.4%)	23 (53.5%)			
Female	30 (52.6%)	20 (46.5)			
**Clinical features**					
Family history	4 (7.0%)	3 (42.9%)	0.99^c^	0.21, 4.69	0.994
Duration (months)^§^	13.0 (8.5, 18.0)	12.0 (8.0, 20.0)	< 0.5^b^	− 3.00, 3.00	0.759
Bulbar onset	14 (24.6%)	21 (48.8%)	2.93^c^	1.25, 6.85	**0.012**
Predominance of LMN dysfunction	32 (56.1%)	31 (72.1%)	2.02^c^	0.87, 4.71	0.102
MRC sum score*	137.85 ± 23.55	128.83 ± 27.99	9.02^a^	− 1.22, 19.26	0.084
ALSFRS-R*	38.12 ± 5.67	34.40 ± 6.82	3.73^a^	1.25, 6.21	**0.004**
ALSFRS-bulbar scores*	9.89 ± 2.34	9.00 ± 2.59	0.90^a^	− 0.09, 1.88	0.073
ALSFRS-respiratory scores*	11.33 ± 1.24	10.53 ± 1.75	0.80^a^	0.17, 1.42	**0.013**
Progression rate^§^	0.75 (0.42, 0.93)	1.00 (0.53, 1.83)	0.33^b^	0.09, 0.65	**0.007**
Progression rate (rapid^#^)	12 (21.1%)	23 (53.5%)	4.31^c^	1.80, 10.34	**0.001**
**Anthropometric measures**					
Weight (kg)*	66.33 ± 12.61	63.80 ± 11.20	2.53^a^	− 2.29, 7.35	0.3
BMI (kg/m^2^)*	24.07 ± 3.14	22.79 ± 2.95	1.28^a^	0.06, 2.51	**0.041**
Fat-free mass (kg)*	48.02 ± 10.29	47.56 ± 9.38	0.46^a^	− 3.51, 4.43	0.819
Muscle mass (kg)	44.89 ± 9.75	44.45 ± 8.88	0.44^a^	− 3.32, 4.20	0.817
Fat mass (kg)*	18.31 ± 5.38	16.24 ± 4.34	2.07^a^	0.08, 4.06	**0.041**
Bone mass (kg)*	3.15 ± 0.55	3.12 ± 0.50	0.02^a^	− 0.19, 0.24	0.82
MM%*	67.57 ± 6.23	69.63 ± 5.71	− 2.07^a^	− 4.48, 0.34	0.092
FM%*	27.67 ± 6.57	25.47 ± 6.05	2.21^a^	− 0.34, 4.75	0.089
Waist hip rate*	0.91 ± 0.08	0.89 ± 0.04	0.02^a^	− 0.005, 0.04	0.119
Visceral fat index*	10.12 ± 2.47	9.34 ± 1.95	0.78^a^	− 0.12, 1.69	0.09
Fat mass of limbs (kg)*	9.17 ± 2.70	8.13 ± 2.17	1.04^a^	0.04, 2.03	**0.042**
Fat mass of trunk (kg)*	9.14 ± 2.68	8.11 ± 2.17	1.03^a^	0.04, 2.03	**0.041**

*Data presented as mean ± standard deviation. ^§^Data presented as median(IQR).

^a^Mean difference. ^b^ median difference. ^c^ Odd ratio.

^#^Rapid disease progression defined as monthly decline of ALSFRS score exceeding 1 score.

*LMN* lower motor neuron, *MRC* Medical Research Council, *ALSFRS-R* revised ALS Functional Rating Scale, *BMI* Body Mass Index, *MM%* proportion of muscle mass, *FM%* proportion of fat mass.

P-value < 0.05 is shown in bold.

**Figure 1:**
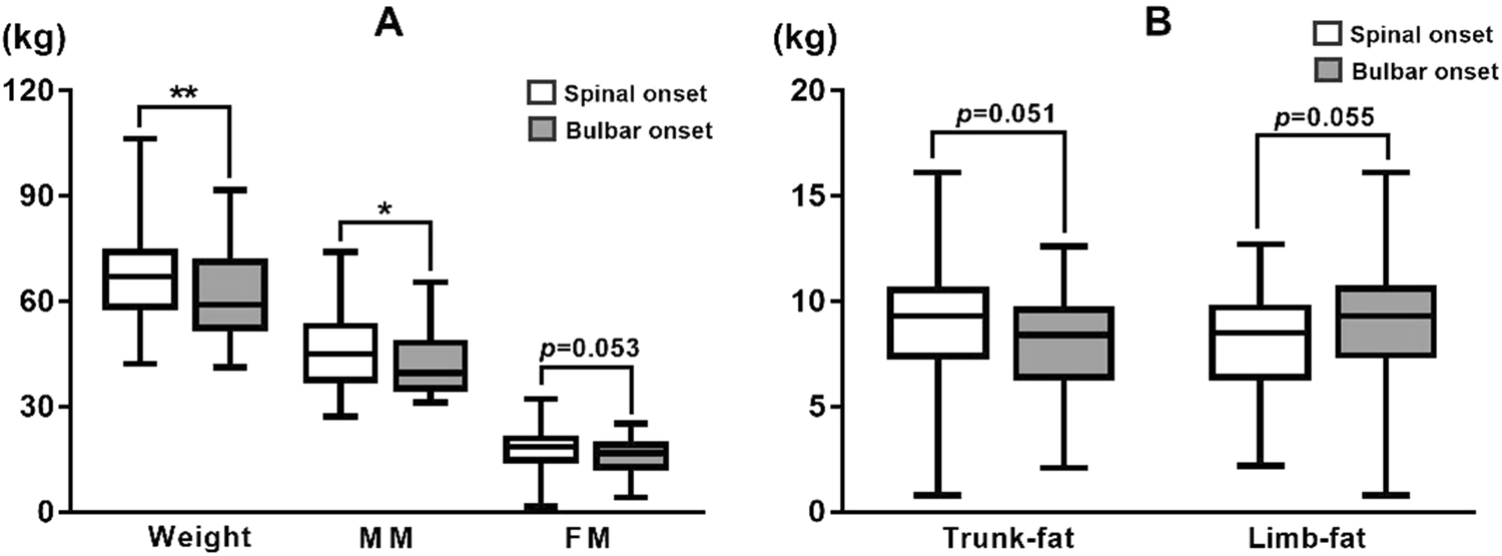
Weight and body composition in ALS patients grouped by site of onset. (**A**) Patients with bulbar onset had a significantly lower weight and MM than those with spinal onset. (**B**) FM in trunk was relatively lower in patients with bulbar onset while FM in limbs was relatively higher in these patients than in those with spinal onset. *MM* muscle mass, *FM* fat mass. *p < 0.05; **p < 0.01.

No significant differences in age, sex, family history, or disease duration were found among patients grouped by weight variation. There were 21(48.8%) bulbar-onset cases in patients with significant weight loss at diagnosis, which was significantly higher than those without significant weight loss (24.6%, p < 0.05). Among these bulbar-onset cases with significant weight loss at diagnosis, 76.2% (16/21) also had spinal involvement at baseline. With regard to body composition, we found that muscle mass (MM), bone mass (BM), WHR, and visceral fat index (VFI) were significantly lower in patients with bulbar onset (Fig. 1A). Interestingly, fat mass in the limbs was relatively higher in these patients than in patients with spinal onset, although the result did not reach significance (Fig. 1B). The predominant involvement of the lower motor neurons occurred in 72.1% of patients with significant weight loss and 56.1% without significant weight loss, while the difference was not statistically significant (p > 0.05). Moreover, no significant difference in body composition was noted between patients with predominant involvement of upper motor neurons and those with predominant involvement of lower motor neurons (p > 0.05).

At diagnosis, patients with significant weight loss had lower ALSFRS-R scores, lower respiratory scores of ALSFRS-R, and a higher rate of disease progression (p < 0.05). Seven patients had a positive family history of ALS, six of whom did genetic screening (4 were *SOD1* mutation, 1 was *FUS* mutation, 1 was *DCTN1* mutation; Supplementary Table 1). No significant difference in nutritional parameters, including weight and body composition, was found between patients with and without a family history, but the negative results were probably due to the small size of patients with a family history. According to the DPR at diagnosis, patients were divided into two groups: slow disease progression (DPR < 1) and fast disease progression (DPR ≥ 1). However, the nutritional parameters also did not differ between patients with slow disease progression and those with rapid disease progression.

Additionally, parameters of body composition varied in the groups with different weight variations (Table 2). The levels of BMI, FM, and FM in limbs and trunk were significantly lower in patients with weight loss exceeding 5% (p < 0.05). Patients with significant weight loss at diagnosis tend to have a relatively higher proportion of muscle mass and a relatively lower body fat percentage (p = 0.092, p = 0.089). Moreover, patients with weight loss ≥ 5% had a significantly lower WHR, VFI, and FM% than those without weight loss (p < 0.05).

The correlation analysis showed that the total ALSFRS-R score was positively correlated with the weight at diagnosis (r = 0.323, p = 0.001), muscle mass (r = 0.267, p = 0.008), fat mass (r = 0.207, p = 0.043), fat in the limbs (r = 0.21, p = 0.04), fat in the trunk (r = 0.204, p = 0.046), and the bone mass (r = 0.267, p = 0.008). Moreover, the correlation analysis revealed a significant correlation between the bulbar score of the ALSFRS-R and the parameters of body fat, including total fat mass (r = 0.237, p = 0.018), fat mass in the limbs (r = 0.238, p = 0.017), VFI (r = 0.237, p = 0.018), and WHR (r = 0.311, p = 0.002). However, no significant correlation was found between the respiratory score of ALSFRS-R and nutritional parameters including weight, BMI, and body composition.

### Short-term change of nutritional parameters

There were 88 patients followed up at 6 months after first visit. During the follow-up period, endpoints occurred in five patients (4 patients died and 1 patient was tracheotomized), and PEG was used in 3 patients. The weight of 6 patients at follow-up was unknown due to their mobility problems and difficulty in standing on their own. Of 77 living patients without tracheotomy, 10.4% (8/77) were underweight at the follow-ups. Weight loss occurred in 52 (67.5%) patients at follow-up, of which 51.9% (27/52) had weight loss exceeding 5% and 26.9% (14/52) had weight loss exceeding 10% comparing with weight at diagnosis. Among patients with weight loss at diagnosis, 36 (54.5%) continued to lose weight as noted at subsequent follow-ups. Onset age, ALSFRS-R’ at follow-ups, and DPR’ at follow-ups were significantly different among patients with weight loss ≥ 5% and those without weight loss ≥ 5% during follow-ups (Supplementary Table 2).

Twenty-one patients reassessed their body composition during outpatient visits, and 14 (66.7%) patients had a weight loss at follow-up. The mean age of onset was 51.19 ± 11.88 years, and 7 (33.3%) patients were bulbar onset. The ALSFRS-R scores were significantly decreased during follow-ups compared with those in diagnosis (Table 3). Of all 21 patients with twice the evaluation of body composition, FFM, and MM values were significantly decreased during follow-ups, while no significant difference was found in FM, WHR, and VFI (Table 3). Among those patients with weight loss during follow-ups, we found that the FFM, MM and FM were significantly decreased during follow-ups (p < 0.01, Supplementary Table 3). Further analysis of changes in fat showed that fat in limbs was significantly decreased while no significant difference was found in WHR and VFI (p < 0.01, Supplementary Table 3). We also investigated the changes in nutritional parameters in patients with weight gain, but no significant difference was found in weight and body composition.

**Table 3:** Comparison of nutritional parameters at diagnosis and follow-up in patients (n = 21).

Parameters	Diagnosis	Follow-up	Effect size ^a^	95% CI	P value
ALSFRS-R	40.71 ± 4.64	36.43 ± 6.40	− 4.29	− 6.03, − 2.54	** < 0.001**
Weight (kg)	66.34 ± 13.85	64.41 ± 14.18	− 1.93	− 4.12, 0.26	0.081
BMI (kg/m^2^)	23.27 ± 3.37	22.76 ± 3.36	− 0.51	− 1.42, 0.40	0.257
FFM (kg)	49.34 ± 10.55	47.81 ± 11.06	− 1.53	− 2.53, − 0.52	**0.005**
MM (kg)	46.12 ± 10.00	44.69 ± 10.48	− 1.43	− 2.38, − 0.48	**0.005**
FM (kg)	17.00 ± 4.99	16.60 ± 5.01	− 0.40	− 2.00, 1.20	0.607
WHR	0.88 ± 0.04	0.89 ± 0.05	0.01	− 0.02, 0.04	0.457
VFI	9.30 ± 2.05	9.49 ± 2.40	0.20	− 0.64, 1.03	0.633
FM%	25.48 ± 5.90	25.70 ± 6.59	0.23	− 1.69, 2.15	0.807
MM%	69.63 ± 5.55	69.39 ± 6.22	− 0.23	− 2.01, 1.54	0.786
Fat in limbs (kg)	8.53 ± 2.50	8.30 ± 2.51	− 0.22	− 1.03, 0.58	0.568

^a^Mean difference.

*ALSFRS-R* revised ALS Functional Rating Scale, *BMI* Body Mass Index, *FFM* fat-free mass, *MM* muscle mass, *FM* fat mass, *WHR* waist hip rate, *VFI* visceral fat index, *FM%* proportion of fat mass, *MM%* proportion of muscle mass.

P-value < 0.05 is shown in bold.

Using the Pearson correlation analysis, we found a significant correlation between weight’ at follow-ups and total ALSFRS-R’ score at follow-ups (r = 0.343, p = 0.002). There was no correlation between total ALSFRS-R’ score at the follow-up and nutritional parameters at diagnosis (Supplementary Table 4). The mean rate of disease progression at follow-ups was 1.0, by which patients were divided into two groups: slow progression (DPR’ < 1) and rapid progression (DPR’ ≥ 1). Clinical features and nutritional parameters were compared between groups (Supplementary Table 5). No significant difference was found in onset age, site of onset, and disease duration between patients with different progression rates. The proportion of patients with weight loss during the follow-up period did not significantly differ between patients with slow or fast disease progression, while patients with weight loss ≥ 5% at follow-ups tend to progress rapidly (p < 0.001). Patients with rapid disease progression during follow-ups had lower VFI, a lower proportion of FM, and a higher proportion of MM at baseline (p < 0.05). In multifactor linear regression, we found that FFM at diagnosis and weight’ during follow-ups were independently correlated with DPR’ at follow-ups after adjusting for other contributing factors (p < 0.05, Table 4).

**Table 4:** Multivariate linear regression analysis of disease progression rate during follow-ups (n = 83).

Variable	OR	95% CI	T	p-value
Onset age	0.001	− 0.026, 0.027	0.045	0.964
Disease duration	− 0.013	− 0.03, 0.004	− 1.547	0.127
ALSFRS-R	− 0.006	− 0.051, 0.039	− 0.276	0.784
ALSFRS-bulbar scores	− 0.003	− 0.117, 0.112	− 0.044	0.965
ALSFRS-respiratory scores	0.051	− 0.124, 0.226	0.578	0.565
FFM	0.067	0.027, 0.106	3.358	**0.001**
FM	− 0.109	− 0.253, 0.036	− 1.502	0.138
VFI	0.277	− 0.041, 0.594	1.737	0.087
Weight’	− 0.04	− 0.073, − 0.006	− 2.362	**0.021**

*ALSFRS-R* revised ALS Functional Rating Scale, *FFM* fat-free mass, *FM* fat mass, *VFI* visceral fat index, *Weight’* weight at follow-ups.

P-value < 0.05 is shown in bold.

## Discussion

Our study found that a large number of Chinese ALS patients (66%) had weight loss at diagnosis, and 43.0% had a weight loss greater than 5%. Weight loss occurred most commonly in patients with the bulbar form of onset and worse neurological function, correlating with faster disease progression. These results are consistent with a large-scale population-based study in Netherlands including 2420 ALS cases, among which 67.5% of patients reported weight loss at diagnosis with a mean loss of weight of 6.2 (9.7)%^14^. As shown in our study, weight loss is a long-term ongoing feature in the course of ALS. Previous studies also reported a loss of weight in ALS patients during different periods even before disease onset, which is associated with a poor prognosis^14–17^. Moglia et al. found that the median survival in patients with a mean monthly weight loss exceeding 1% at diagnosis was less than half that in those who gained weight^4^. Marin et al.^3^ also identified a nearly 30% and 34% increased risk of death with each 5% weight loss at diagnosis and follow-up, respectively.

The exact mechanism of weight loss remains largely unclear. Investigation of body composition may offer insights to address this issue. Previous studies with small sample sizes^18,19^ found that ALS patients have a lower BMI and FFM than healthy controls, but no significant difference in body composition was found among patients and controls in this study. The inconsistent results might be explained by the different disease duration of patients and the demographic differences of controls. Although muscle and fat were both lower in ALS patients than in controls in this study, the difference in parameters of body composition did not reach significance at the early stage of the disease. Ngo et al.^5^ also did not find significant differences in body composition between 62 patients with ALS and 45 healthy controls, while the study didn’t match age among patients and controls. Moreover, the difference in techniques for measuring body composition may also lead to different results. In our study, the difference in fat mass is relatively higher between patients and controls among parameters of body composition, which may reach statistical significance as the disease progresses or by expanding the sample size. In longitudinal observation, Nau et al.^11^ reported a loss of lean mass while gaining FM over 6 months, with weight loss and energy storage. Marin et al.^3^ also suggested a significant decrease in weight, BMI, and lean mass with increased FM and triceps skinfold thickness during follow-up. We also found that FFM and FM decreased in patients with weight loss as the disease progressed, which is consistent with previous studies, while the FM also decrease in our patients with weight loss and FM did not significantly increase in our patients with weight gain. The contradictory findings might be explained by the difference in energy expenditure and calorie intake. Nau et al.^11^ observed a loss of lean mass and increased fat mass, resulting in weight loss, while the energy store was increased in ALS patients. When comparing intake with consumption, no difference was found at the beginning, while calorie intake was higher than expenditure during follow-ups. Based on that study, we speculated that when patients consumed more calories than their expenditure, residual energy would be stored in the form of fat, maintaining body weight. In contrast, insufficient intake resulted in fat burning for energy, similar to the patients in our study. This hypothesis could also be indirectly supported by Ngo’s study^5^, which found that FM decreased significantly in ALS patients with loss of appetite during the 18-month follow-up but increased in patients with intact appetite.

Body fat might be the major factor involved in energy metabolism resulting in weight variation in ALS patients. A study by Barone et al.^20^ found that FM was significantly lower in underweight patients and it increased in patients with a higher BMI, while no significant difference was found in FFM. This study also observed lower BMI and FM in patients with significant weight loss. Impaired cellular energy homeostasis and mitochondrial dysfunction have been considered one of the most important mechanisms in ALS^21^. As one of the major nutrients, fat plays an important role in the energy supplementation of ALS patients. Evidence from mutant *SOD1* mice suggests a preferential lipid-based energy metabolism in muscle fibers at a presymptomatic stage, which is independent of motor neuron degeneration^22,23^. The underlying mechanisms of metabolic changes might be related to altered protein function in metabolic pathways, including glycolysis and β-oxidation^22^, which could probably be restored by a high-fat diet^24,25^. Our study found patients with a lower proportion of FM tend to progress rapidly during follow-up. Lee et al.^26^ also suggested that loss of fat is correlated with faster disease progression in ALS patients (n = 20), indicating the positive effects of fat in ALS. Consistently, Park et al.^27^ found longer survival in ALS patients (n = 53) with an increased body fat rate. Future studies with larger sample sizes are needed to explore the effect of body fat on the prognosis of ALS patients.

Fat metabolism might be distinctive in different parts of the body in ALS patients. Lindauer et al.^13^ identified remarkably increased visceral fat and unremarkably decreased subcutaneous fat in ALS patients compared with controls, indicating the tendency of visceral adipose tissues depot in ALS patients. Our study found fat in limbs significantly decreased in patients with weight loss during follow-ups, while no significant difference was found in VFI or WHR, suggesting the predominant wasting of subcutaneous adipose tissues (SAT) in ALS patients. This specific pattern of lipid metabolism and storage may be explained by the abnormalities in the energy metabolism of skeletal muscle^28^ and probable neural denervation of SAT^29^ in the pathological process of ALS. The other alternative interpretation is the physiological differences between SAT and VAT^30^, including the stronger insulin resistance of VAT^31,32^, different sensitivity to lipolysis, and different expression of adipokines^33^. Moreover, survival analysis from Lindauer et al.^13^ indicated that increased subcutaneous fat rather than visceral fat was associated with a better prognosis. Other studies also indicated an association between increased triceps skinfold thickness and a better outcome^3^. Our study also found that patients with lower fat mass in limbs or trunk tend to have a relatively higher rate of progression during follow-ups; however, the result was only statistically significant in the association between VFI and disease progression rate at follow-ups. Moreover, the effect of VFI on disease progression rate did not reach statistical significance after adjusting for other covariates. It is widely accepted that visceral fat is a predictor of poor prognosis in a variety of diseases^34,35^, and there has been little studies on the effect of visceral fat and subcutaneous fat on the prognosis of ALS patients. This study observed a trend for increased rate of disease progression in patients with lower visceral fat, but this finding needs to be interpreted with caution due to the relatively small sample size, and more large-sample studies are needed to confirm and explore the findings in the future.

The current study has a number of limitations. First, patients with restricted mobility or difficulty standing on their own were excluded due to the limitations of measurements with the body composition analyser, probable leading to exclusion of patients with a progressed course of ALS. Second, caloric intake and energy expenditure might affect the changes in weight and body composition. Considering the possible effect of cognitive function on diet and nutrition^36^, the lack of cognitive assessment in this study would also affect the results. In order to correct the effect of diet on the rate of disease progression during the follow-up period, we included patients' weight during the follow-up period in the multivariate regression analysis. There is a wide variety of foods in China and the type of food eaten by Chinese people also varies from region to region. Until now, there is no unified scale for the dietary assessment of ALS patients in China, and we are further exploring and discussing this part. We hope to further explore the influence of diet on morbidity, weight maintenance, and prognosis of ALS patients in the future. Third, this study did not include data from the pulmonary function test (FVC%) to study the effect of respiratory function on nutritional status. Instead of FVC% in the pulmonary function test, we used the respiratory scores of ALSFRS-R to analyse the correlation between nutritional state and respiratory status^37,38^, which may affect our results. Moreover, we observed the change of nutritional state in a short period (6 months) and study the effect of nutrition on patients' functional progression. We intend to follow these patients for a long-term period to observe the impact of these nutritional parameters on survival in the future.

## Conclusion

In conclusion, we found that weight loss was common in the course of ALS with the wasting of muscle and fat, correlating with rapid disease progression. Body composition has potential prognostic value for ALS, and fat may have a potential protective role in ALS patients. Future large-scale research could focus on fat metabolism to provide detailed individual dietary guidelines to delay the progression of the disease.

## Methods

### Participants

We performed a study of 100 consecutive patients presented at Peking Union Medical College Hospital from October 2020 to April 2021. All patients were newly diagnosed with clinically definite, probable, or laboratory-supported probable ALS according to the revised El Escorial criteria^39^. Exclusion criteria included acute infections, malignant tumours, untreated or uncontrolled endocrine diseases, other malnutrition disorders, and inability to accomplish the anthropometric measurements. Additionally, we included 50 healthy controls for comparison of body composition. This study was approved by the Ethics Committee of Clinical Research of Peking Union Medical College Hospital (Beijing, China) (approval number: JS-2624). All methods were performed in accordance with the Declaration of Helsinki and STROBE guidelines. All participants provided signed informed consent.

### Clinical features and nutritional parameters at baseline and follow-ups

Demographic and clinical data were collected at the first meeting, including sex, age of onset, site of onset, disease duration (from onset to diagnosis), predominant involvement of the upper motor neurons or lower motor neurons^40,41^, and revised ALS functional rating scale (ALSFRS-R). Muscle strength was assessed by the UK Medical Research Council (MRC), and the total strength was calculated by summing the MRC scores of the muscle groups. The rate of disease progression (DPR) at diagnosis was calculated using the formula: DPR = (48-ALSFRS-R)/disease duration (months). We defined rapid disease progression as DPR ≥ 1 according to previous studies^42,43^.

Anthropometric characteristics were measured at the time of recruitment, including height, weight, WHR, and detailed body composition. Height was accessed when patients stood in an upright position. Weight before disease onset was reported from patients or family members. Weight, WHR, and body composition at diagnosis were evaluated by a body composition analyser (TongFang Health Technology, Beijing, China) using the direct segmental multifrequency bioelectrical impedance analysis method (DSM-BIA). The models for body composition were developed based on the results of body composition measured by isotope dilution, magnetic resonance imaging (MRI), and dual-energy X-ray absorptiometry (DEXA)^44^:

1 $$Y = 1.072Xe^{{ - \left( {\frac{{k - 1.786}}{{1.77}}} \right)^{2} }} + 0.3194K^{2} - 2.073K + 3.713$$

X represents the impedance index; when K = 1, 2, and 3, Y represents total body water (TBW), fat mass (FM), and bone mass, respectively.

2 $${\text{Protien mass}} = 1.01 \times \left( {{\text{BW}} - {\text{FM}} - {\text{BM}} - {\text{TBW}}} \right) + 1.29$$

$${\text{FFM}} = {\text{BW}} + {\text{TBW}} + {\text{Protienmass}}$$

$${\text{Muscle Mass}} = {\text{TBW}} + {\text{Protienmass}}$$

The parameters of body composition included FFM, MM, FM, visceral fat index (VFI), and percentage of body mass (FM%, MM%). BMI was calculated using the formula: BMI = weight/height^2^ (kg/m^2^).

All patients were followed 6 months after the first visit through a face-to-face evaluation or telephone contact. By telephone call, we collected patients’ weights at follow-ups (Weight’) by self-report from patients who followed our directions with the same scale. Moreover, ALSFRS-R scores were re-evaluated at follow-ups by outpatient visits or telephone follow-ups. The DPR during follow-ups was calculated using the formula: DPR’ = (ALSFRS-R at diagnosis—ALSFRS-R during follow-ups)/6 (months). During clinic follow-ups, weight and body composition of patients were reassessed using the body composition analyzer. Change of each nutritional parameter at different time points was observed and compared. The percentage of weight loss was calculated by comparing the weight from different points in time (before disease onset, at baseline, and during follow-ups). A percentage exceeding 5% was defined as significant weight loss^3,14,45^.

### Statistical analysis

The Statistical Package for the Social Sciences software (SPSS, Version 22) was used for the data analysis. Categorical variables were expressed as frequencies (proportions) and analysed by the chi-square test or Fisher’s exact test. Normally distributed continuous variables such as age, weight, and BMI were expressed as the mean ± SD and compared by independent Student’s t-tests. Non-normally distributed continuous variables, including most parameters of body composition, are presented as the median (interquartile range, IQR) and were analysed using the Mann–Whitney test between groups. The associations between clinical parameters and nutritional parameters were analysed using Spearman correlations. Multivariate logistic regression was performed to identify factors associated with weight loss at diagnosis. The short-term outcome was evaluated by ALSFRS-R at follow-up. A multiple linear regression model was constructed to investigate the correlation of clinical features and nutritional state at diagnosis with ALSFRS-R score at follow-up. The differences in weight and body composition between baseline and follow-ups were analyzed by paired t-test. The results with a p value < 0.05 were considered to be significant.

## Supplementary Information

Supplementary Information.

## Data Availability

The datasets used or analysed during the current study available from the corresponding author on reasonable request.
