# Supplementary material for: Correlation of weight and body composition with disease progression rate in patients with amyotrophic lateral sclerosis
Source: Sci Rep. 2022 Aug 2;12:13292. doi: 10.1038/s41598-022-16229-9 (PMC9345931; doi:10.1038/s41598-022-16229-9)
Supplement: Supplementary file 1 — Supplementary Information. [file 41598_2022_16229_MOESM1_ESM.docx]

**Table 1.** Nutritional parameters among FALS patients with different genetic mutations

|  | *SOD1* mutation  (n=4) | *DCTN1* mutation  (n=1) | *FUS* mutation  (n=1) |
| --- | --- | --- | --- |
| Weight (kg) | 64.85±12.87 | 85.6 | 64.6 |
| BMI (kg/m^2^) | 23.3±3.81 | 27 | 25.2 |
| Fat-free mass (kg) | 48.3±9.86 | 64.1 | 40.2 |
| Muscle Mass (kg) | 45.15±9.31 | 60.1 | 37.5 |
| Fat Mass (kg) | 16.55±5.16 | 21.5 | 24.4 |
| Bone Mass (kg) | 3.16±0.53 | 4.01 | 2.73 |
| Waist hip rate | 0.87±0.02 | 0.92 | 0.97 |
| Visceral fat index | 8.85±1.60 | 10.4 | 13.3 |

All the parameters in *SOD1* mutation were presented as mean ± standard deviation.

**Table 2.** Clinical features in patients stratified by weight changes during follow-ups

|  | Significant weight loss  (n=27) | Insignificant weight loss  (n=46) | Effect size | 95% CI | P value |
| --- | --- | --- | --- | --- | --- |
| Age at onset (years)* | 55.07±8.75 | 49.07±10.22 | 6.009 | 1.32, 10.70 | **0.013** |
| Sex (male) | 11(40.7%) | 21(45.7%) | 0.818 | 0.31, 2.14 | 0.683 |
| Family history | 3(11.1%) | 3(6.5%) | 0.558 | 0.10, 2.98 | 0.498 |
| Duration (months)^§^ | 12(7, 17) | 13(10, 21) | 3.000 | -1.00, 7.00 | 0.125 |
| Bulbar onset | 13(48.1%) | 13(28.3%) | 0.424 | 0.16, 1.14 | 0.087 |
| Predominance of LMN dysfunction | 19(70.4%) | 26(56.5%) | 0.547 | 0.20, 1.50 | 0.24 |
| ALSFRS-R* | 35.48±6.20 | 37.24±6.22 | -1.758 | -4.76, 1.25 | 0.247 |
| ALSFRS-R'* | 26.26±8.33 | 33.85±8.17 | -7.589 | -11.57, -3.61 | **<0.001** |
| DPR^§^ | 1.00(0.64, 1.75) | 0.74(0.42, 0.96) | -0.316 | -0.60, -0.08 | **0.013** |
| DPR'^§^ | 1.17(0.83, 2.33) | 0.50(0, 0.83) | -0.833 | -1.17, -0.33 | **<0.001** |

*Data presented as mean ± standard deviation. §Data presented as median(IQR).

LMN, lower motor neuron; ALSFRS-R, revised ALS Functional Rating Scale at diagnosis; ALSFRS-R’, revised ALS Functional Rating Scale at follow-ups; DPR, disease progression rate at diagnosis; DPR’, disease progression rate at follow-ups.

P-value <0.05 is shown in bold.

**Table 3.** Comparison of nutritional parameters at diagnosis and follow-up in patients with weight loss (n=14)

| Parameters | Diagnosis | Follow-up | Effect size^a^ | 95% CI | P value |
| --- | --- | --- | --- | --- | --- |
| ALSFRS-R | 40.14±5.36 | 35.36±6.92 | -4.79 | -7.09, -2.49 | 0.001 |
| Weight (kg) | 67.85±13.68 | 63.36±13.33 | -4.49 | -6.07, -2.90 | **<0.001** |
| BMI (kg/m^2^) | 23.79±3.63 | 22.26±3.45 | -1.53 | -2.10, -0.96 | **<0.001** |
| FFM (kg) | 49.97±9.88 | 47.37±9.82 | -2.6 | -3.34, -1.86 | **<0.001** |
| MM (kg) | 46.71±9.37 | 44.26±9.31 | -2.45 | -3.15, -1.75 | **<0.001** |
| FM (kg) | 17.88±5.40 | 15.99±5.26 | -1.89 | -3.16, -0.62 | **0.007** |
| WHR | 0.89±0.03 | 0.89±0.05 | -0.002 | -0.04, 0.03 | 0.898 |
| VFI | 9.54±2.18 | 9.17±2.65 | -0.37 | -1.15, 0.40 | 0.32 |
| FM% | 25.97±6.23 | 24.84±6.88 | -1.14 | -2.62, 0.35 | 0.123 |
| MM% | 69.18±5.82 | 70.19±6.44 | 1.01 | -0.36, 2.38 | 0.134 |
| Fat in limbs (kg) | 8.97±2.70 | 8.00±2.63 | -0.97 | -1.60, -0.34 | **0.005** |

All the parameters in SOD1 mutation were presented as mean ± standard deviation.

^a^ mean difference.

ALSFRS-R, revised ALS Functional Rating Scale; BMI, Body Mass Index; FFM, fat-free mass; MM, muscle mass; FM, fat mass; WHR, waist hip rate; VFI, visceral fat index; FM%, proportion of fat mass; MM%, proportion of muscle mass.

P-value <0.05 is shown in bold.

**Table 4.** Correlation analysis between nutritional parameters at baseline and clinical features

| p value (r) | ALSFRS-R' | DPR' |
| --- | --- | --- |
| Weight | 0.303(0.115) | 0.498(0.075) |
| BMI | 0.429(0.088) | 0.795(-0.029) |
| FFM | 0.627(0.054) | 0.115(0.174) |
| MM | 0.625(0.054) | 0.11(0.177) |
| FM | 0.5(0.075) | 0.294(-0.117) |
| BM | 0.638(0.052） | 0.11(0.177) |
| MM% | 0.744(0.036) | 0.063(0.205) |
| FM% | 0.782(-0.031） | 0.068(-0.202) |
| WHR | 0.913(0.012) | 0.592(0.06) |
| VFI | 0.793(0.029) | 0.381(-0.098) |
| Weight' | **0.002(0.343)** | 0.351(-0.108) |

ALSFRS-R’, revised ALS Functional Rating Scale at follow-ups; DPR’, disease progression rate at follow-ups; BMI, Body Mass Index; FFM, fat-free mass; MM, muscle mass; FM, fat mass; BM, bone mass; FM%, proportion of fat mass; MM%, proportion of muscle mass; WHR, waist hip rate; VFI, visceral fat index; Weight, weight at baseline; Weight’, weight at follow-ups.

P-value <0.05 is shown in bold.

**Table 5.** Clinical features and nutritional parameters among patients with different rate of progression during follow-ups

|  | Slow progression  (n=49) | Rapid progression  (n=34) | Effect size | 95% CI | P value |
| --- | --- | --- | --- | --- | --- |
| Age at onset (yrs)* | 51.51±10.28 | 50.94±11.37 | 0.57^a^ | -4.20, 5.34 | 0.813 |
| Sex |  |  | 0.54^c^ | 0.23, 1.32 | 0.176 |
| Male | 20(40.8%) | 19(55.9%) |  |  |  |
| Female | 29(59.2%) | 15(44.1%) |  |  |  |
| Duration (months)^§^ | 14(9, 20) | 13(8, 10) | -1.5^b^ | -5.00, 2.00 | 0.404 |
| Bulbar onset | 16(32.7%) | 12(35.3%） | 1.13^c^ | 0.45, 2.83 | 0.802 |
| Progression rate^§^ | 0.77(0.41, 1.09) | 0.76(0.43, 1.15) | 0.02^b^ | -0.18, 0.25 | 0.774 |
| **Nutritional parameters** |  |  |  |  |  |
| Weight (kg)* | 64.61±10.63 | 65.40±13.15 | -0.78^a^ | -5.99, 4.42 | 0.766 |
| BMI (kg/m^2^)* | 23.79±2.68 | 23.17±3.33 | 0.63^a^ | -0.69, 1.94 | 0.347 |
| Fat-free mass (kg)* | 46.43±9.21 | 49.29±10.73 | -2.85^a^ | -7.23, 1.52 | 0.198 |
| Muscle Mass (kg)* | 43.38±8.73 | 46.08±10.16 | -2.71^a^ | -6.85, 1.44 | 0.198 |
| Fat Mass (kg)* | 18.18±4.27 | 16.11±5.61 | 2.07^a^ | -0.09, 4.23 | 0.06 |
| Bone Mass (kg)* | 3.06±0.49 | 3.22±0.58 | -0.15^a^ | -0.39, 0.08 | 0.196 |
| MM%* | 66.95±5.51 | 70.47±6.59 | -3.52^a^ | -6.17, -0.87 | **0.01** |
| FM%* | 28.31±5.81 | 24.59±6.95 | 3.73^a^ | 0.93, 6.53 | **0.01** |
| Waist hip rate* | 0.90±0.05 | 0.90±0.09 | <0.01^a^ | -0.03, 0.03 | 0.953 |
| Visceral fat index* | 10.11±1.92 | 9.13±2.54 | 0.98^a^ | 0.003, 1.95 | **0.049** |
| Fat mass of limbs (kg)* | 9.11±2.14 | 8.06±2.81 | 1.05^a^ | -0.03, 2.13 | 0.057 |
| Fat mass of trunk (kg)* | 9.08±2.13 | 8.05±2.80 | 1.02^a^ | -0.05, 2.10 | 0.062 |
| Weight'(kg)* | 63.99±10.34 | 59.65±11.27 | 4.34^a^ | -0.62, 9.31 | 0.085 |
| Weight loss during follow-ups | 17(37.0%) | 8(25.8%) | 0.593^c^ | 0.22, 1.62 | 0.305 |
| Weight loss ≥ 5% during follow-ups | 7(15.9%) | 20(69.0%) | 0.085^c^ | 0.03, 0.26 | **<0.001** |

*Data presented as mean ± standard deviation. ^§^Data presented as median(IQR).

^a^ mean difference. ^b^ median difference. ^c^ Odd ratio.
FM%, proportion of fat mass; MM%, proportion of muscle mass; Weight, weight at baseline; Weight’, weight at follow-ups.

P-value <0.05 is shown in bold.
